# Supplementary material for: Prostate-specific membrane antigen positron emission tomography compared to multiparametric MRI for prostate cancer diagnosis: a protocol for a systematic review and meta-analysis
Source: BMJ Open. 2021 Dec 10;11(12):e052277. doi: 10.1136/bmjopen-2021-052277 (PMC8666885; doi:10.1136/bmjopen-2021-052277)
Supplement: Author's manuscript [file bmjopen-2021-052277.draft_revisions.pdf]

BMJ Open

BMJ Open is committed to open peer review. As part of this commitment we make the peer review history of every article we publish publicly available.

When an article is published we post the peer reviewers' comments and the authors' responses online. We also post the versions of the paper that were used during peer review. These are the versions that the peer review comments apply to.

The versions of the paper that follow are the versions that were submitted during the peer review process. They are not the versions of record or the final published versions. They should not be cited or distributed as the published version of this manuscript.

BMJ Open is an open access journal and the full, final, typeset and author-corrected version of record of the manuscript is available on our site with no access controls, subscription charges or pay-per-view fees (http://bmjopen.bmj.com).

If you have any questions on BMJ Open's open peer review process please email info.bmjopen@bmj.com

BMJ Open

Prostate-specific membrane antigen positron emission tomography compared to multiparametric magnetic resonance imaging for prostate cancer diagnosis: a protocol for a systematic review and meta-analysis

Journal:	*BMJ Open*
Manuscript ID	bmjopen-2021-052277
Article Type:	Protocol
Date Submitted by the Author:	14-Apr-2021

Complete List of Authors:	Zhao, Yi; Imperial College London,<br>Morka, Naomi; University College London Medical School,<br>Simpson, Benjamin Scott; University College London, UCL Division of Surgery & Interventional Science<br>Freeman, Alex; University College Hospital London, Department of Histopathology<br>Kirkham, Alex; University College London Hospitals NHS Foundation Trust, Department of Radiology<br>Kelly, Daniel; Cardiff University, School of Healthcare Sciences, College of Biomedical and Life Sciences<br>Whitaker, Hayley; University College London, UCL Division of Surgery and Interventional Science<br>Emberton, Mark; University College London, Division of Surgery and Interventional Sciences; University College London Hospital, Department of Urology<br>Norris, Joseph; University College London, UCL Division of Surgery & Interventional Science

Keywords:	Urological tumours < ONCOLOGY, Prostate disease < UROLOGY, Magnetic resonance imaging < RADIOLOGY & IMAGING

SCHOLARONE™
Manuscripts

I, the Submitting Author has the right to grant and does grant on behalf of all authors of the Work (as defined in the below author licence), an exclusive licence and/or a non-exclusive licence for contributions from authors who are: i) UK Crown employees; ii) where BMJ has agreed a CC-BY licence shall apply, and/or iii) in accordance with the terms applicable for US Federal Government officers or employees acting as part of their official duties; on a worldwide, perpetual, irrevocable, royalty-free basis to BMJ Publishing Group Ltd ("BMJ") its licensees and where the relevant Journal is co-owned by BMJ to the co-owners of the Journal, to publish the Work in this journal and any other BMJ products and to exploit all rights, as set out in our licence.

The Submitting Author accepts and understands that any supply made under these terms is made by BMJ to the Submitting Author unless you are acting as an employee on behalf of your employer or a postgraduate student of an affiliated institution which is paying any applicable article publishing charge ("APC") for Open Access articles. Where the Submitting Author wishes to make the Work available on an Open Access basis (and intends to pay the relevant APC), the terms of reuse of such Open Access shall be governed by a Creative Commons licence – details of these licences and which Creative Commons licence will apply to this Work are set out in our licence referred to above.

Other than as permitted in any relevant BMJ Author's Self Archiving Policies, I confirm this Work has not been accepted for publication elsewhere, is not being considered for publication elsewhere and does not duplicate material already published. I confirm all authors consent to publication of this Work and authorise the granting of this licence.

For peer review only

Prostate-specific membrane antigen positron emission tomography compared to multiparametric magnetic resonance imaging for prostate cancer diagnosis: a protocol for a systematic review and meta-analysis

Yi Zhao[1], Naomi Morka[2], Benjamin S. Simpson[3], Alex Freeman[4], Alex Kirkham[5] , Daniel Kelly[6], Hayley C. Whitaker[7], Mark Emberton[7,8] Joseph M. Norris[7,8]

ME and JMN are joint senior authors.

[1] Imperial College London School of Medicine, London, UK

[2] UCL Medical School, University College London, London, UK

[3] UCL Cancer Institute, University College London, London, UK

[4] Department of Pathology, University College London Hospitals NHS Foundation Trust, London, UK

[5] Department of Radiology, University College London Hospitals NHS Foundation Trust, London, UK

[6] School of Healthcare Sciences, Cardiff University, Wales, UK

[7] UCL Division of Surgery & Interventional Science, University College London, London, UK

[8] Department of Urology, University College London Hospitals NHS Foundation Trust, London, UK

Correspondence to: Yi Zhao;

Imperial College London

For peer review only - http://bmjopen.bmj.com/site/about/guidelines.xhtml

Exhibition Rd, South Kensington, London SW7 2BU

yi.zhao18@imperial.ac.uk

Ethics: No ethical considerations apply.

Word count: 1,988 words

For peer review only

ABSTRACT

Introduction: The introduction of multiparametric magnetic resonance imaging (mpMRI) has improved almost every aspect of the prostate cancer diagnostic pathway. However, the novel imaging technique, prostate-specific membrane antigen positron emission tomography (PSMA PET) may have demonstrable accuracy in detecting and staging prostate cancer. Here, we describe a protocol for a systematic review and meta-analysis comparing mpMRI to PSMA PET for the diagnosis of suspected prostate cancer.

Methods and Analysis: A systematic search of MEDLINE, EMBASE, Pubmed and Cochrane databases will be conducted. The Preferred Reporting Items for Systematic Reviews and Meta-Analyses (PRISMA) guidelines will be followed for screening, data extraction, statistical analysis, and reporting. Included papers will be full-text articles providing original data, written in English articles and comparing the use of PSMA PET with mpMRI in the diagnosis of prostate cancer. All studies published between July 1977 and March 2021 will be eligible for inclusion. Study bias and quality will be assessed using a modified Newcastle-Ottawa score. To ensure methodological rigour, this protocol is written following the PRISMA Protocol (PRISMA-P) 2015 checklist.

For peer review only - http://bmjopen.bmj.com/site/about/guidelines.xhtml

Ethics and dissemination: Ethical approval will not be required for this systematic review. Findings will be disseminated through peer-reviewed publications and presentations at both national and international conferences.

PROSPERO registration number: CRD42021239296.

Strengths and limitations of this study:

- This will be the first comprehensive systematic review and meta-analysis of the use of PSMA PET and mpMRI in the detection of prostate cancer, written in line with PRISMA guidelines.
- This study will use comprehensive statistical methods to minimise the effect of heterogeneity in the literature on the determined results.
- The heterogeneity of study cohorts may limit the strength of conclusions drawn.
- There is likely to be a less extensive analysis of this focus due to the limited number of studies focusing on the primary diagnosis.
- Due to the recent introduction of PSMA PET as a mean of primary diagnosis, there may be limited long term data on the clinical outcomes associated with this technology.

BACKGROUND

Multiparametric magnetic resonance imaging (mpMRI) is now routinely used in the pre-biopsy risk stratification of suspected prostate cancer, affording significantly improved diagnostic accuracy over traditional techniques.[1] [2]

For peer review only

For peer review only - http://bmjopen.bmj.com/site/about/guidelines.xhtml

However, approximately 10-20% of clinically significant prostate tumours may be overlooked when using mpMRI,[3] [4] and as a result, the search for a more specific and sensitive imaging modality continues.

Prostate-specific membrane antigen positron emission tomography (PSMA PET) has primarily been used as a staging tool for established prostate cancer, however, there has been a recent surge in interest in using PSMA PET for diagnosis.[5] PSMA PET enables radionuclide imaging of PSMA, a type II transmembrane glycoprotein, overexpressed in the vast majority of prostate tumours[6],[7] and is known to correlate with high serum levels of prostate-specific antigen and a higher Gleason score, potentially making this a more targeted and specific marker of prostate cancer.[8] Conventionally, 68Ga-PSMA PET is coupled with either Computed tomography (CT) imaging or MRI,[8] however, alternative radionuclides including, Flurone-18, have been proposed to improve imaging quality by overcoming the limitations exhibited when using the 68Ga radionuclides.[9]

Early PSMA PET diagnostic evaluation studies have shown promising results in the diagnosis of prostate cancer, particularly when compared to the current gold standard imaging approach, mpMRI. It is time to collate the growing evidence in this field, comparing the characteristics of these two imaging modalities. This systematic review aims to analyse and summarise the diagnostic accuracies of PSMA PET and mpMRI for the first time, to potentially elucidate whether an alternative but reliable imaging modality can be determined for the detection of prostate cancer.

For peer review only - http://bmjopen.bmj.com/site/about/guidelines.xhtml

METHODS AND ANALYSIS

This planned systematic review protocol is written in line with the Preferred Reporting Items for Systematic Reviews and Meta-Analyses Protocol (PRISMA) 2015 Checklist.[10] Included studies will undergo analysis and thematic synthesis to derive statistical data comparing the sensitivity, specificity, positive predictive value (PPV) and negative predictive value (NPV) between PSMA PET and mpMRI.

Search methodology

A systematic search will be carried out using the MEDLINE, PubMed, EMBASE and Cochrane databases to retrieve all studies that contribute relevant evidence. The search will include Medical Subject Heading (MESH) terms and free text with appropriate Boolean operators. The following terms will be included in the search: ‘’prostate’’, ‘’cancer’’, ‘’diagnosis’’, as well as multiple synonyms for the term ‘’PSMA PET’’ and ‘’mpMRI’’ to account for differences in terminology. All eligible studies published between July 1977 to Feb 2021 will be uploaded to Rayyan, a semiautomated tool aimed at improving the speed and reporting accuracy of articles during the initial screening process. [11] To identify any missed studies or additional data, a further manual search of references in all included articles will be performed. In the case of absent or ambiguous data, the corresponding authors will be contacted directly for clarification.

Study selection and data extraction

For peer review only - http://bmjopen.bmj.com/site/about/guidelines.xhtml

Three researchers will screen eligible studies independently and assess titles and abstracts for relevance. Should an article be considered eligible, the full text will be retrieved for further review of eligibility. Any disagreements between reviewers will be discussed until a consensus is reached, or a fourth reviewer will be consulted. All exclusions will be noted for further analysis and the reasons documented in detail to generate the PRISMA flow diagram.

Inclusion criteria

To be included in the analysis, studies must compare the diagnostic accuracies between PSMA PET and mpMRI modality for prostate cancer. Comparisons are focused on investigating sensitivities, specificities, PPVs and NPVs.

Exclusion criteria

Correspondence articles, expert opinions, conference abstracts, review articles and case reports will be excluded. Selected studies must be written in the English language. Studies that do not make a direct comparison between PSMA PET and mpMRI, such as those investigating the combined accuracy in the diagnosis, will be excluded. Articles that solely focus on the diagnostic accuracy of PSMA PET or mpMRI alone will also be removed.

Data extraction

All desired data will be collated in a dedicated datasheet. All reviewers will extract data independently.[12] Table 1 summarises the data to be collected.

For peer review only - http://bmjopen.bmj.com/site/about/guidelines.xhtml
For peer review only

Table 1 Data collection items

Item no.	Data title	Data type
1	Year of publication	Study characteristic
2	Study authors	Study characteristic
3	Study design	Study characteristic
4	Patient population	Demographics
5	Number of participants	Demographics
6	mpMRI scoring scheme used	Methodology
7	Definition for clinically significant disease	Methodology
8	Definition for lesion visibility and invisibility	Methodology
9	Sample processing approach	Methodology
10	PSMA PET technique and radionuclide used	Outcome
11	Differential quantification of accuracies	Outcome

mpMRI, multiparametric MRI; no, number

For peer review only - http://bmjopen.bmj.com/site/about/guidelines.xhtml

Endpoints

The primary endpoint will be statistically significant differences in quantitative measurements (e.g. sensitivity, specificity, PPV and NPV) determining diagnostic accuracy between PSMA PET and mpMRI. We will focus on key themes within the literature such as MRI scoring system (eg, Prostate Imaging-Reporting and Data System (PI-RADS), Likert, and radiogenomic features) and the criteria used to define lesion visibility (PI-RADS or Likert score thresholds).

Meta-analysis

The pooled quantitative diagnostic accuracy values between PSMA PET and mpMRI will be compared in the meta-analysis. In the first instance, sensitivity and specificity values will be retrieved or calculated. If studies do not provide these values they will be calculated from clinical tables or requested from study authors. In the instance of a substantial proportion of articles using any other metric, these values may also be retrieved and calculated separately. The distribution of untransformed, logit and double-arcsine transformed proportions will be assessed for normality using the Shapiro-Wilk test and density plots. The set of ratios that most resemble a normal distribution will be used for the combined analysis.

Inter-study variation and heterogeneity will be quantified through I^2 and as the true effect size for all studies are unlikely to be identical (due to methodological differences, institutional differences, interpretation differences) a random-effects model will be fitted for estimation and estimated with partial pooling. Once the model is fit, leave-one-out analyses (LOO) and accompanying diagnostic plots will be used to identify influential studies and will be quantified using externally

For peer review only - http://bmjopen.bmj.com/site/about/guidelines.xhtml

studentised residuals, different in fits values, Cook's distances, covariance ratios, LOO estimates of the amount of heterogeneity, LOO values of the test statistics for heterogeneity, hat values and weights. Each study will be removed one at a time, and the summary proportions will be re-estimated based on the remaining n-1 studies. Studies with a statistically significant influence on the fitted model will be removed as outliers and the model re-fitted. Once heterogeneity has been minimised and outliers removed, summary estimates will be compared between PSMA PET and mpMRI. All data analysis and visualisation will be performed via the R statistical environment (version 3.6.1, 2019-07-05) using the "metafor" and "meta" packages.

Risk of bias in individual studies

A modified Newcastle-Ottawa score, designed to assess observational cohort studies, will be used to assess bias and quality across the selected studies.[11] The scoring system is split into three main sections: selection, comparability, and outcome. Within each section, there are sub-questions that assess the quality of research methodology at the study level. Three reviewers will be independently involved in this process with any disagreements settled by consensus. The outcome of the bias assessment will be used to influence data synthesis by assessing the applicability and reliability of the data produced. Studies may be excluded if found to be of low quality or suggesting high levels of bias. If they are included, appropriate commentaries will follow in the discussion. As this review is focused on the diagnostic accuracies of prostate cancer between PSMA PET and mpMRI, we will modify non-applicable sections of the scoring systems to reflect the nature of the evidence base and reduce reporting inaccuracy as reported previously.[12]

For peer review only - http://bmjopen.bmj.com/site/about/guidelines.xhtml

Patient and public involvement

There will be no patient and public involvement in this study.

For peer review only

For peer review only - http://bmjopen.bmj.com/site/about/guidelines.xhtml

DISCUSSION

Today, mpMRI is now widely adopted for pre-biopsy risk stratification in men with suspected prostate cancer and has been incorporated into national and international prostate cancer guidelines.[13] [14] [15] However, the utility of alternative imaging modalities in this setting is under-explored. Through this systematic review, we aim to compare the diagnostic accuracies of a novel imaging technique, PSMA PET, against the new standard-of-care, mpMRI. The summation of evidence in this fledgeling field will aid in clinical and research decision-making when considering pooled accuracy in the diagnosis of primary prostate cancer, so benefitting patient care.

So far, the bulk of clinical evidence for PSMA PET has supported the use of this technique in the staging of prostate cancer. Indeed, PSMA PET/CT has been shown to independently predict treatment response to salvage radiation treatment, whilst PSMA PET/MRI has demonstrable accuracy in the staging of primary diagnosed prostate cancer.[16] [17] Furthermore, the two most frequently used radionuclides, 68Ga or 18F, have been shown to have similar results in the staging of clinically significant prostate cancer.[18] However, only in recent years, has considerable attention been given to the potential benefits of PSMA PET in the diagnosis of suspected prostate cancer.

This systematic review will combine the extant data, comparing the diagnostic performance of PSMA PET and mpMRI in this novel yet growing field, for the first time. The collation and analysis of these data will allow a better understanding of the differences in diagnostic utility between PSMA PET and mpMRI. Additionally, this process will also aid in clinical decision making for prostate cancer diagnosis as well as potential areas for further research.

Trial status

For peer review only - http://bmjopen.bmj.com/site/about/guidelines.xhtml
For peer review only

- Preliminary searches: Started
- Piloting of the study selection process: Started
- Formal screening: not started
- Data extraction: not started
- Risk of bias assessment: not started
- Data analysis: not started

Draft search strategy

((((Prostat* AND (Cancer OR malignan* OR adenocarcinoma OR lesion* OR Disease)) AND (PSMA OR "prostate-specific membrane antigen positron emission tomography")) AND (MR OR magnetic resonance imaging OR MP-MRI OR multi-parametric MRI OR multi-parametric magnetic resonance imaging OR multiparametric MRI OR "multiparametric magnetic resonance imaging")) AND Diagnosis).ti,ab

ETHICS AND DISSEMINATION

Due to the nature of this review, there are no relevant ethical concerns and informed consent will not be required. The protocol and systematic review will be disseminated via a peer-reviewed journal.

For peer review only

Author Affiliations

[1] Imperial College London School of Medicine, London, UK

[2] UCL Medical School, University College London, London, UK

[3] UCL Cancer Institute, University College London, London, UK

[4] Department of Pathology, University College London Hospitals NHS Foundation Trust, London, UK

[5] Department of Radiology, University College London Hospitals NHS Foundation Trust, London, UK

[6] School of Healthcare Sciences, Cardiff University, Wales, UK

[7] UCL Division of Surgery & Interventional Science, University College London, London, UK

[8] Department of Urology, University College London Hospitals NHS Foundation Trust, London, UK

Email: Yi Zhao: yi.zhao18@ic.ac.uk, Naomi Morka: naomi.morka.17@ucl.ac.uk, Benjamin S. Simpson: b.simpson@ucl.ac.uk, Alex Freeman: alex.freeman2@nhs.net, Alex Kirkam: alexkirkham@nhs.net, Daniel Kelly: kellydm@cardiff.ac.uk, Hayley C. Whitaker: hayley.whitaker@ucl.ac.uk, Mark Emberton: m.emberton@ucl.ac.uk, Joseph M. Norris: joseph.norris@ucl.ac.uk

Twitter Naomi Morka @naomi_morka, Benjamin S. Simpson @b3nsimp, Daniel Kelly @profdkelly, Hayley C. Whitaker @HayleyWhitaker, Mark Emberton @EmbertonMark, Joseph M. Norris @MrJosephNorris

For peer review only

Contributors The authors' contribution includes, but is not limited to, the following: NM, BSS and JMN drafted the manuscript and created the study concept. AK, AF, DK, HCW and ME provided supervision and guidance during the study. All authors reviewed and approved the manuscript in its current form. YZ is the guarantor of this work.

Funding Norris is funded by the Medical Research Council (MRC) (MR/S00680X/1). Simpson is funded by the Royal Marsden Cancer Charity. Emberton receives research support from the United Kingdom's National Institute of Health Research (NIHR) UCLH/UCL Biochemical Research Centre.

Competing Interests Norris receives funding from the MRC. Simpson receives funding from the Royal Marsden Cancer Charity. Whitaker receives funding from Prostate Cancer UK, the Urology Foundation and Rosetrees Trust. Kirkham, Freeman and Emberton have stock interest in Nuada Medical Ltd. Emberton acts as a consultant, trainer and proctor to Sonatherm Inc; Angiodynamics Inc; Exact Imaging Inc.

Patient consent for publication Not required

Provenance and peer review Not commissioned; externally peer-reviewed

Open access This is an open-access article distributed in accordance with the Creative Commons Attribution 4.0 Unreported (CC BY 4.0) license which permits others to copy, redistribute, remix, transform and build upon this work for any purpose, provided the original work is properly cited, a link to the licence is given, and indication of whether changes were made. See https://creativecommons.org/licenses/by/4.0/.

For peer review only

ORCID IDs

- Yi Zhao https://orcid.org/0000-0002-4563-4344
- Naomi Morka https://orcid.org/0000-0002-1925-7223
- Joseph M. Norris http://orcid.org/0000-0003-2294-0303
- Benjamin S. Simpson http://orcid.org/0000-0003-3685-6110
- Daniel Kelly http://orcid.org/0000-0002-1847-0655
- Hayley C. Whitaker https://orcid.org/0000-0002-2695-0202

For peer review only

REFERENCES

1 Ahmed HU, Bosaily AE-S, Brown LC, *et al.* Diagnostic accuracy of multi-parametric MRI and TRUS biopsy in prostate cancer (PROMIS): a paired validating confirmatory study. *The Lancet* 2017;**389**:815–22. doi:10.1016/S0140-6736(16)32401-1

2 Kasivisvanathan V, Rannikko AS, Borghi M, *et al.* MRI-Targeted or Standard Biopsy for Prostate-Cancer Diagnosis. *N Engl J Med* 2018;**378**:1767–77. doi:10.1056/NEJMoa1801993

3 Bass EJ, Pantovic A, Connor M, *et al.* A systematic review and meta-analysis of the diagnostic accuracy of biparametric prostate MRI for prostate cancer in men at risk. *Prostate Cancer Prostatic Dis* 2020;:1–16. doi:10.1038/s41391-020-00298-w

4 Radtke JP, Kuru TH, Boxler S, *et al.* Comparative analysis of transperineal template saturation prostate biopsy versus magnetic resonance imaging targeted biopsy with magnetic resonance imaging-ultrasound fusion guidance. *J Urol* 2015;**193**:87–94. doi:10.1016/j.juro.2014.07.098

5 Satapathy S, Singh H, Kumar R, *et al.* Diagnostic Accuracy of 68Ga-PSMA PET/CT for Initial Detection in Patients With Suspected Prostate Cancer: A Systematic Review and Meta-Analysis. *Am J Roentgenol* 2021;**216**:599–607. doi:10.2214/AJR.20.23912

6 Wright GL, Haley C, Beckett ML, *et al.* Expression of prostate-specific membrane antigen in normal, benign, and malignant prostate tissues. *Urol Oncol Semin Orig Investig* 1995;**1**:18–28. doi:10.1016/1078-1439(95)00002-Y

7 Bouchelouche K, Choyke PL. Advances in PSMA Positron Emission Tomography (PET) of Prostate Cancer. *Curr Opin Oncol* 2018;**30**:189–96. doi:10.1097/CCO.0000000000000439

8 Hoffmann MA, Wieler HJ, Baues C, *et al.* The Impact of 68Ga-PSMA PET/CT and PET/MRI on the Management of Prostate Cancer. *Urology* 2019;**130**:1–12. doi:10.1016/j.urology.2019.04.004

9 Kesch C, Kratochwil C, Mier W, *et al.* 68Ga or 18F for Prostate Cancer Imaging? *J Nucl Med* 2017;**58**:687–8. doi:10.2967/jnumed.117.190157

10 Moher D, Shamseer L, Clarke M, *et al.* Preferred reporting items for systematic review and meta-analysis protocols (PRISMA-P) 2015 statement. *Syst Rev* 2015;**4**:1. doi:10.1186/2046-4053-4-1

11 Wells G, Shea B, Oconnell D. The Newcastle-Ottawa scale (NOS) for assessing the quality of nonrandomized studies in meta-analyses. Available: ohri.ca/programs/clinical_epidemiology/oxford.asp

12 Genetic landscape of prostate cancer conspicuity on multiparametric MRI: a protocol for a systematic review and bioinformatic analysis | BMJ Open. https://bmjopen.bmj.com/content/10/1/e034611.abstract (accessed 1 Mar 2021).

For peer review only

13 Jones R, Cornford P. EAU and NICE guidelines for the diagnosis and management of prostate cancer. How wide is the channel? *J Clin Urol* 2018;**11**:149–53. doi:10.1177/2051415817720353

14 Dasgupta P, Davis J, Hughes S. NICE guidelines on prostate cancer 2019. *BJU Int* 2019;**124**:1–1. doi:https://doi.org/10.1111/bju.14815

15 Wise J. NICE recommends MRI for suspected prostate cancer to reduce biopsies. *BMJ* 2018;**363**:k5290. doi:10.1136/bmj.k5290

16 Emmett L, Leeuwen PJ van, Nandurkar R, *et al.* Treatment Outcomes from 68Ga-PSMA PET/CT–Informed Salvage Radiation Treatment in Men with Rising PSA After Radical Prostatectomy: Prognostic Value of a Negative PSMA PET. *J Nucl Med* 2017;**58**:1972–6. doi:10.2967/jnumed.117.196683

17 Grubmüller B, Baltzer P, Hartenbach S, *et al.* PSMA Ligand PET/MRI for Primary Prostate Cancer: Staging Performance and Clinical Impact. *Clin Cancer Res Off J Am Assoc Cancer Res* 2018;**24**:6300–7. doi:10.1158/1078-0432.CCR-18-0768

18 Kuten J, Fahoum I, Savin Z, *et al.* Head-to-Head Comparison of 68Ga-PSMA-11 with 18F-PSMA-1007 PET/CT in Staging Prostate Cancer Using Histopathology and Immunohistochemical Analysis as a Reference Standard. *J Nucl Med Off Publ Soc Nucl Med* 2020;**61**:527–32. doi:10.2967/jnumed.119.234187

For peer review only

For peer review only

For peer review only - http://bmjopen.bmj.com/site/about/guidelines.xhtml

Reporting checklist for protocol of a systematic review and meta analysis.

Based on the PRISMA-P guidelines.

Instructions to authors

Complete this checklist by entering the page numbers from your manuscript where readers will find each of the items listed below.

Your article may not currently address all the items on the checklist. Please modify your text to include the missing information. If you are certain that an item does not apply, please write "n/a" and provide a short explanation.

Upload your completed checklist as an extra file when you submit to a journal.

In your methods section, say that you used the PRISMA-Preporting guidelines, and cite them as:

Moher D, Shamseer L, Clarke M, Ghersi D, Liberati A, Petticrew M, Shekelle P, Stewart LA. Preferred Reporting Items for Systematic Review and Meta-Analysis Protocols (PRISMA-P) 2015 statement. Syst Rev. 2015;4(1):1.

		Reporting Item	Page Number
**Title**			
Identification	#1a	Identify the report as a protocol of a systematic review	1
Update	#1b	If the protocol is for an update of a previous systematic review, identify as such	N/A This protocol is not an update
**Registration**			
	#2	If registered, provide the name of the registry (such as PROSPERO) and registration number	3
**Authors**			
Contact	#3a	Provide name, institutional affiliation, e-mail address of all protocol authors; provide physical mailing address of corresponding author	1, 11
Contribution	#3b	Describe contributions of protocol authors and identify the guarantor of the review	11

For peer review only

Amendments

	#4	If the protocol represents an amendment of a previously completed or published protocol, identify as such and list changes; otherwise, state plan for documenting important protocol amendments	N/A. It is not an amendment

Support

Sources	#5a	Indicate sources of financial or other support for the review	11
Sponsor	#5b	Provide name for the review funder and / or sponsor	N/A Review is not funded
Role of sponsor or funder	#5c	Describe roles of funder(s), sponsor(s), and / or institution(s), if any, in developing the protocol	N/A Review is not funded

Introduction

Rationale	#6	Describe the rationale for the review in the context of what is already known	4
Objectives	#7	Provide an explicit statement of the question(s) the review will address with reference to participants, interventions, comparators, and outcomes (PICO)	4

Methods

Eligibility criteria	#8	Specify the study characteristics (such as PICO, study design, setting, time frame) and report characteristics (such as years considered, language, publication status) to be used as criteria for eligibility for the review	5
Information sources	#9	Describe all intended information sources (such as electronic databases, contact with study authors, trial registers or other grey literature sources) with planned dates of coverage	5
Search strategy	#10	Present draft of search strategy to be used for at least one electronic database, including planned limits, such that it could be repeated	10
Study records - data management	#11a	Describe the mechanism(s) that will be used to manage records and data throughout the review	5
Study records -	#11b	State the process that will be used for selecting studies (such	5

For peer review only

selection process		as two independent reviewers) through each phase of the review (that is, screening, eligibility and inclusion in meta-analysis)	
Study records - data collection process	#11c	Describe planned method of extracting data from reports (such as piloting forms, done independently, in duplicate), any processes for obtaining and confirming data from investigators	5
Data items	#12	List and define all variables for which data will be sought (such as PICO items, funding sources), any pre-planned data assumptions and simplifications	6
Outcomes and prioritization	#13	List and define all outcomes for which data will be sought, including prioritization of main and additional outcomes, with rationale	7
Risk of bias in individual studies	#14	Describe anticipated methods for assessing risk of bias of individual studies, including whether this will be done at the outcome or study level, or both; state how this information will be used in data synthesis	8
Data synthesis	#15a	Describe criteria under which study data will be quantitatively synthesised	7
Data synthesis	#15b	If data are appropriate for quantitative synthesis, describe planned summary measures, methods of handling data and methods of combining data from studies, including any planned exploration of consistency (such as I2, Kendall's τ)	7
Data synthesis	#15c	Describe any proposed additional analyses (such as sensitivity or subgroup analyses, meta-regression)	7
Data synthesis	#15d	If quantitative synthesis is not appropriate, describe the type of summary planned	N/A Quantitative synthesis is appropriate
Meta-bias(es)	#16	Specify any planned assessment of meta-bias(es) (such as publication bias across studies, selective reporting within studies)	7
Confidence in cumulative evidence	#17	Describe how the strength of the body of evidence will be assessed (such as GRADE)	7

For peer review only

Notes:

- 1b: N/A This protocol is not an update
- 4: N/A. It is not an amendment
- 5b: N/A Review is not funded
- 5c: N/A Review is not funded
- 15d: N/A Quantitative synthesis is appropirate The PRISMA-P elaboration and explanation paper is distributed under the terms of the Creative Commons Attribution License CC-BY. This checklist was completed on 07. April 2021 using https://www.goodreports.org/, a tool made by the EQUATOR Network in collaboration with Penelope.ai

For peer review only

Prostate-specific membrane antigen positron emission tomography compared to multiparametric magnetic resonance imaging for prostate cancer diagnosis: a protocol for a systematic review and meta-analysis

Journal:	*BMJ Open*
Manuscript ID	bmjopen-2021-052277.R1
Article Type:	Protocol
Date Submitted by the Author:	23-Oct-2021
Complete List of Authors:	Zhao, Yi; Imperial College London,<br>Morka, Naomi; University College London Medical School,<br>Simpson, Benjamin Scott; University College London, UCL Division of Surgery & Interventional Science<br>Freeman, Alex; University College Hospital London, Department of Histopathology<br>Kirkham, Alex; University College London Hospitals NHS Foundation Trust, Department of Radiology<br>Kelly, Daniel; Cardiff University, School of Healthcare Sciences, College of Biomedical and Life Sciences<br>Whitaker, Hayley; University College London, UCL Division of Surgery and Interventional Science<br>Emberton, Mark; University College London, Division of Surgery and Interventional Sciences; University College London Hospital, Department of Urology<br>Norris, Joseph; University College London, UCL Division of Surgery & Interventional Science
**Primary Subject Heading**:	Urology
Secondary Subject Heading:	Oncology, Diagnostics
Keywords:	Urological tumours < ONCOLOGY, Prostate disease < UROLOGY, Magnetic resonance imaging < RADIOLOGY & IMAGING

SCHOLARONE™ Manuscripts

For peer review only - http://bmjopen.bmj.com/site/about/guidelines.xhtml

I, the Submitting Author has the right to grant and does grant on behalf of all authors of the Work (as defined in the below author licence), an exclusive licence and/or a non-exclusive licence for contributions from authors who are: i) UK Crown employees; ii) where BMJ has agreed a CC-BY licence shall apply, and/or iii) in accordance with the terms applicable for US Federal Government officers or employees acting as part of their official duties; on a worldwide, perpetual, irrevocable, royalty-free basis to BMJ Publishing Group Ltd ("BMJ") its licensees and where the relevant Journal is co-owned by BMJ to the co-owners of the Journal, to publish the Work in this journal and any other BMJ products and to exploit all rights, as set out in our licence.

The Submitting Author accepts and understands that any supply made under these terms is made by BMJ to the Submitting Author unless you are acting as an employee on behalf of your employer or a postgraduate student of an affiliated institution which is paying any applicable article publishing charge ("APC") for Open Access articles. Where the Submitting Author wishes to make the Work available on an Open Access basis (and intends to pay the relevant APC), the terms of reuse of such Open Access shall be governed by a Creative Commons licence – details of these licences and which Creative Commons licence will apply to this Work are set out in our licence referred to above.

Other than as permitted in any relevant BMJ Author's Self Archiving Policies, I confirm this Work has not been accepted for publication elsewhere, is not being considered for publication elsewhere and does not duplicate material already published. I confirm all authors consent to publication of this Work and authorise the granting of this licence.

For peer review only

Prostate-specific membrane antigen positron emission tomography compared to multiparametric magnetic resonance imaging for prostate cancer diagnosis: a protocol for a systematic review and meta-analysis

Yi Zhao[1], Naomi Morka[2], Benjamin S. Simpson[3], Alex Freeman[4], Alex Kirkham[5] , Daniel Kelly[6], Hayley C. Whitaker[7], Mark Emberton[7,8] Joseph M. Norris[7,8]

ME and JMN are joint senior authors.

[1] Imperial College London School of Medicine, London, UK

[2] UCL Medical School, University College London, London, UK

[3] UCL Cancer Institute, University College London, London, UK

[4] Department of Pathology, University College London Hospitals NHS Foundation Trust, London, UK

[5] Department of Radiology, University College London Hospitals NHS Foundation Trust, London, UK

[6] School of Healthcare Sciences, Cardiff University, Wales, UK

[7] UCL Division of Surgery & Interventional Science, University College London, London, UK

[8] Department of Urology, University College London Hospitals NHS Foundation Trust, London, UK

Correspondence to: Yi Zhao;

Imperial College London

For peer review only

Exhibition Rd, South Kensington, London SW7 2BU

yi.zhao18@imperial.ac.uk

Ethics: No ethical considerations apply.

Word count: 1,988 words

For peer review only

ABSTRACT

Introduction: The introduction of multiparametric magnetic resonance imaging (mpMRI) has improved almost every aspect of the prostate cancer diagnostic pathway. However, the novel imaging technique, prostate-specific membrane antigen positron emission tomography (PSMA PET) may have demonstrable accuracy in detecting and staging prostate cancer. Here, we describe a protocol for a systematic review and meta-analysis comparing mpMRI to PSMA PET for the diagnosis of suspected prostate cancer.

Methods and Analysis: A systematic search of MEDLINE, EMBASE, Pubmed and Cochrane databases will be conducted. The Preferred Reporting Items for Systematic Reviews and Meta-Analyses (PRISMA) guidelines will be followed for screening, data extraction, statistical analysis, and reporting. Included papers will be full-text articles providing original data, written in English articles and comparing the use of PSMA PET with mpMRI in the diagnosis of prostate cancer. All studies published between July 1977 and March 2021 will be eligible for inclusion. Study bias and quality will be assessed using a modified Newcastle-Ottawa score. To ensure the quality of the reporting of studies, this protocol is written following the PRISMA Protocol (PRISMA-P) 2015 checklist.

Ethics and dissemination: Ethical approval will not be required for this systematic review. Findings will be disseminated through peer-reviewed publications and presentations at both national and international conferences.

PROSPERO registration number: CRD42021239296.

Strengths and limitations of this study:

- This will be the first comprehensive systematic review and meta-analysis of the use of PSMA PET and mpMRI in the detection of prostate cancer, written in line with PRISMA guidelines.
- This study will use comprehensive statistical methods to minimise the effect of heterogeneity in the literature on the determined results.
- The heterogeneity of study cohorts may limit the strength of conclusions drawn.
- There is likely to be a less extensive analysis of this focus due to the limited number of studies focusing on the primary diagnosis.
- Due to the recent introduction of PSMA PET as a mean of primary diagnosis, there may be limited long term data on the clinical outcomes associated with this technology.

BACKGROUND

Multiparametric magnetic resonance imaging (mpMRI) is now routinely used in the pre-biopsy risk stratification of suspected prostate cancer, affording significantly improved diagnostic accuracy over traditional techniques.[1] [2]

For peer review only - http://bmjopen.bmj.com/site/about/guidelines.xhtml

However, approximately 10-20% of clinically significant prostate tumours may be overlooked when using mpMRI,[3] [4] and as a result, the search for a more specific and sensitive imaging modality continues.

Prostate-specific membrane antigen positron emission tomography (PSMA PET) has primarily been used as a staging tool for established prostate cancer, however, there has been a recent surge in interest in using PSMA PET for diagnosis.[5] PSMA PET enables radionuclide imaging of PSMA, a type II transmembrane glycoprotein, overexpressed in the vast majority of prostate tumours, [6] [7] and is known to correlate with high serum levels of prostate-specific antigen and a higher Gleason score, potentially making this a more targeted and specific marker of prostate cancer.[8] Conventionally, 68Ga-PSMA PET is coupled with either Computed tomography (CT) imaging or MRI, [8] however, alternative radionuclides including, Flurone-18, have been proposed to improve imaging quality by overcoming the limitations exhibited when using the 68Ga radionuclides.[9]

Early PSMA PET diagnostic evaluation studies have shown promising results in the diagnosis of prostate cancer, particularly when compared to the current gold standard imaging approach, mpMRI. It is time to collate the growing evidence in this field, comparing the characteristics of these two imaging modalities, which has not been seen in the existing literature. Currently, existing literature focused on the diagnostic performance of PSMA PET and mpMRI in investigating the recurrence and metastasis of prostate cancer.[10] [11] Other studies have focused on the individual diagnostic performance of either PSMA PET or mpMRI. [12]

This systematic review aims to analyse and summarise the diagnostic accuracies of PSMA PET and mpMRI for the first time, to potentially elucidate whether an alternative but reliable imaging modality can be determined for the detection of prostate cancer.

For peer review only - http://bmjopen.bmj.com/site/about/guidelines.xhtml

METHODS AND ANALYSIS

This planned systematic review protocol is written in line with the Preferred Reporting Items for Systematic Reviews and Meta-Analyses Protocol (PRISMA) 2015 Checklist.[13] Included studies will undergo analysis and thematic synthesis to derive statistical data comparing the sensitivity, specificity, positive predictive value (PPV) and negative predictive value (NPV) between PSMA PET and mpMRI. The pooled sensitivities and specificities across the studies will be calculated first before deriving PPV and NPV values.

Search methodology

A systematic search will be carried out using the MEDLINE, PubMed, EMBASE and Cochrane databases to retrieve all studies that contribute relevant evidence. The search will include Medical Subject Heading (MESH) terms and free text with appropriate Boolean operators. The following terms will be included in the search: ‘’prostate’’, ‘’cancer’’, ‘’diagnosis’’, as well as multiple synonyms for the term ‘’PSMA PET’’ and ‘’mpMRI’’ to account for differences in terminology. All eligible studies published between July 1977 to Feb 2021 will be uploaded to Rayyan, a semiautomated tool aimed at improving the speed and reporting accuracy of articles during the initial screening process.[14] To identify any missed studies or additional data, a further manual search of references in all included articles will be performed. In the case of absent or ambiguous data, the corresponding authors will be contacted directly for clarification.

Study selection and data extraction

For peer review only - http://bmjopen.bmj.com/site/about/guidelines.xhtml

Three researchers will screen eligible studies independently and assess titles and abstracts for relevance. Should an article be considered eligible, the full text will be retrieved for further review of eligibility. Any disagreements between reviewers will be discussed until a consensus is reached, or a fourth reviewer will be consulted. All exclusions will be noted for further analysis and the reasons documented in detail to generate the PRISMA flow diagram.

Inclusion criteria

To be included in the analysis, studies must compare the diagnostic accuracies between PSMA PET and mpMRI modality for prostate cancer. Comparisons are focused on investigating sensitivities, specificities, PPVs and NPVs.

Exclusion criteria

Correspondence articles, expert opinions, conference abstracts, review articles and case reports will be excluded. Selected studies must be written in the English language. Studies that do not make a direct comparison between PSMA PET and mpMRI, such as those investigating the combined accuracy in the diagnosis, will be excluded. Articles that solely focus on the diagnostic accuracy of PSMA PET or mpMRI alone will also be removed.

Data extraction

All desired data will be collated in a dedicated datasheet. All reviewers will extract data independently.[15] Table 1 summarises the data to be collected.

For peer review only - http://bmjopen.bmj.com/site/about/guidelines.xhtml

Table 1 Data collection items		
Item no.	Data title	Data type
1	Year of publication	Study characteristic
2	Study authors	Study characteristic
3	Study design	Study characteristic
4	Patient population	Demographics
5	Number of participants	Demographics
6	mpMRI scoring scheme used	Methodology
7	Definition for clinically significant disease	Methodology
8	Definition for lesion visibility and invisibility	Methodology
9	Sample processing approach	Methodology
10	PSMA PET technique and radionuclide used	Methodology

For peer review only - http://bmjopen.bmj.com/site/about/guidelines.xhtml

**12**	Histopathological reference standard (including prostate biopsy and wholemount radical prostetcomy)	Methodology
**11**	Differential quantification of accuracies	Outcome

mpMRI, multiparametric MRI; no, number

For peer review only

Endpoints

The primary endpoint will be statistically significant differences in quantitative measurements (e.g. sensitivity, specificity, PPV and NPV) determining diagnostic accuracy between PSMA PET and mpMRI. We will focus on key themes within the literature such as MRI scoring system (eg, Prostate Imaging-Reporting and Data System (PI-RADS), Likert, and radiogenomic features) and the criteria used to define lesion visibility (PI-RADS or Likert score thresholds).

Meta-analysis

The pooled quantitative diagnostic accuracy values between PSMA PET and mpMRI will be compared in the meta-analysis. In the first instance, sensitivity and specificity values will be retrieved or calculated. If studies do not provide these values they will be calculated from clinical tables or requested from study authors. In the instance of a substantial proportion of articles using any other metric, these values may also be retrieved and calculated separately. The distribution of untransformed, logit and double-arcsine transformed proportions will be assessed for normality using the

For peer review only - http://bmjopen.bmj.com/site/about/guidelines.xhtml

Shapiro-Wilk test and density plots. The set of ratios that most resemble a normal distribution will be used for the combined analysis.

Inter-study variation and heterogeneity will be quantified through I^2 and as the true effect size for all studies are unlikely to be identical (due to methodological differences, institutional differences, interpretation differences) a random-effects model will be fitted for estimation and estimated with partial pooling. Once the model is fit, leave-one-out analyses (LOO) and accompanying diagnostic plots will be used to identify influential studies and will be quantified using externally studentised residuals, different in fits values, Cook's distances, covariance ratios, LOO estimates of the amount of heterogeneity, LOO values of the test statistics for heterogeneity, hat values and weights. Each study will be removed one at a time, and the summary proportions will be re-estimated based on the remaining n-1 studies. Studies with a statistically significant influence on the fitted model will be removed as outliers and the model re-fitted. Once heterogeneity has been minimised and outliers removed, summary estimates will be compared between PSMA PET and mpMRI. All data analysis and visualisation will be performed via the R statistical environment (version 3.6.1, 2019-07-05) using the "mada" and "mvmeta" packages.

Risk of bias in individual studies

The Quadas-2 score will be used to assess bias and quality across the selected studies. [16] The scoring system is split into three main sections: selection, comparability, and outcome. Within each section, there are sub-questions that assess the quality of research methodology at the study level. Three reviewers will be independently involved in this process with any disagreements settled by consensus. The outcome of the bias assessment will be used to influence data synthesis by

For peer review only - http://bmjopen.bmj.com/site/about/guidelines.xhtml

assessing the applicability and reliability of the data produced. Studies may be excluded if found to be of low quality or suggesting high levels of bias. If they are included, appropriate commentaries will follow in the discussion. As this review is focused on the diagnostic accuracies of prostate cancer between PSMA PET and mpMRI, we will modify non-applicable sections of the scoring systems to reflect the nature of the evidence base and reduce reporting inaccuracy as reported previously.[15]

For peer review only

Patient and public involvement

There will be no patient and public involvement in this study.

For peer review only - http://bmjopen.bmj.com/site/about/guidelines.xhtml

DISCUSSION

Today, mpMRI is now widely adopted for pre-biopsy risk stratification in men with suspected prostate cancer and has been incorporated into national and international prostate cancer guidelines.[17] [18] [19] However, the utility of alternative imaging modalities in this setting is under-explored. Through this systematic review, we aim to compare the diagnostic accuracies of a novel imaging technique, PSMA PET, against the new standard-of-care, mpMRI. The summation of evidence in this fledgeling field will aid in clinical and research decision-making when considering pooled accuracy in the diagnosis of primary prostate cancer, so benefitting patient care.

So far, the bulk of clinical evidence for PSMA PET has supported the use of this technique in the staging of prostate cancer. Indeed, PSMA PET/CT has been shown to independently predict treatment response to salvage radiation treatment, whilst PSMA PET/MRI has demonstrable accuracy in the staging of primary diagnosed prostate cancer.[20] [21] Furthermore, the two most frequently used radionuclides, 68Ga or 18F, have been shown to have similar results in the staging of clinically significant prostate cancer.[22] However, only in recent years, has considerable attention been given to the potential benefits of PSMA PET in the diagnosis of suspected prostate cancer.

There may be several limitations in this review. There may be a small number of studies retrieved as the use of PSMA PET is relatively new. Moreover, there may be a small number of centres have access to PSMA PET technology, giving a limited generalisability of our study. The meta-analysis may be affected by the heterogeneity of results reported in each study. The different type of imaging modality used for both PSMA PET and mpMRI may act as confounders for the results.

For peer review only

This systematic review will combine the extant data, comparing the diagnostic performance of PSMA PET and mpMRI in this novel yet growing field, for the first time. The collation and analysis of these data will allow a better understanding of the differences in diagnostic utility between PSMA PET and mpMRI. Additionally, this process will also aid in clinical decision making for prostate cancer diagnosis as well as potential areas for further research.

Trial status

- Preliminary searches: Started
- Piloting of the study selection process: Started
- Formal screening: not started
- Data extraction: not started
- Risk of bias assessment: not started
- Data analysis: not started

Draft search strategy

((((Prostat* AND (Cancer OR malignan* OR adenocarcinoma OR lesion* OR Disease)) AND (PSMA OR "prostate-specific membrane antigen positron emission tomography")) AND (MR OR magnetic resonance imaging OR MP-MRI OR multi-parametric MRI OR multi-parametric magnetic resonance imaging OR multiparametric MRI OR "multiparametric magnetic resonance imaging")) AND Diagnosis).ti,ab

For peer review only

ETHICS AND DISSEMINATION

Due to the nature of this review, there are no relevant ethical concerns and informed consent will not be required. The protocol and systematic review will be disseminated via a peer-reviewed journal.

For peer review only

For peer review only - http://bmjopen.bmj.com/site/about/guidelines.xhtml

Author Affiliations

[1] Imperial College London School of Medicine, London, UK

[2] UCL Medical School, University College London, London, UK

[3] UCL Cancer Institute, University College London, London, UK

[4] Department of Pathology, University College London Hospitals NHS Foundation Trust, London, UK

[5] Department of Radiology, University College London Hospitals NHS Foundation Trust, London, UK

[6] School of Healthcare Sciences, Cardiff University, Wales, UK

[7] UCL Division of Surgery & Interventional Science, University College London, London, UK

[8] Department of Urology, University College London Hospitals NHS Foundation Trust, London, UK

Email: Yi Zhao: yi.zhao18@ic.ac.uk, Naomi Morka: naomi.morka.17@ucl.ac.uk, Benjamin S. Simpson: b.simpson@ucl.ac.uk, Alex Freeman: alex.freeman2@nhs.net, Alex Kirkam: alexkirkham@nhs.net, Daniel Kelly: kellydm@cardiff.ac.uk, Hayley C. Whitaker: hayley.whitaker@ucl.ac.uk, Mark Emberton: m.emberton@ucl.ac.uk, Joseph M. Norris: joseph.norris@ucl.ac.uk

Twitter Naomi Morka @naomi_morka, Benjamin S. Simpson @b3nsimp, Daniel Kelly @profdkelly, Hayley C. Whitaker @HayleyWhitaker, Mark Emberton @EmbertonMark, Joseph M. Norris @MrJosephNorris

For peer review only

Contributors The authors’ contribution includes, but is not limited to, the following: NM, BSS and JMN drafted the manuscript and created the study concept. AK, AF, DK, HCW and ME provided supervision and guidance during the study. All authors reviewed and approved the manuscript in its current form. YZ is the guarantor of this work.

Funding Norris is funded by the Medical Research Council (MRC) (MR/S00680X/1). Simpson is funded by the Royal Marsden Cancer Charity. Emberton receives research support from the United Kingdom’s National Institute of Health Research (NIHR) UCLH/UCL Biochemical Research Centre.

Competing Interests Norris receives funding from the MRC. Simpson receives funding from the Royal Marsden Cancer Charity. Whitaker receives funding from Prostate Cancer UK, the Urology Foundation and Rosetrees Trust. Kirkham, Freeman and Emberton have stock interest in Nuada Medical Ltd. Emberton acts as a consultant, trainer and proctor to Sonatherm Inc; Angiodynamics Inc; Exact Imaging Inc.

Patient consent for publication Not required

Provenance and peer review Not commissioned; externally peer-reviewed

Open access This is an open-access article distributed in accordance with the Creative Commons Attribution 4.0 Unreported (CC BY 4.0) license which permits others to copy, redistribute, remix, transform and build upon this work for any purpose, provided the original work is properly cited, a link to the licence is given, and indication of whether changes were made. See https://creativecommons.org/licenses/by/4.0/.

For peer review only

ORCID IDs

- Yi Zhao https://orcid.org/0000-0002-4563-4344
- Naomi Morka https://orcid.org/0000-0002-1925-7223
- Joseph M. Norris http://orcid.org/0000-0003-2294-0303
- Benjamin S. Simpson http://orcid.org/0000-0003-3685-6110
- Daniel Kelly http://orcid.org/0000-0002-1847-0655
- Hayley C. Whitaker https://orcid.org/0000-0002-2695-0202

For peer review only

REFERENCES

1 Ahmed HU, Bosaily AE-S, Brown LC, *et al.* Diagnostic accuracy of multi-parametric MRI and TRUS biopsy in prostate cancer (PROMIS): a paired validating confirmatory study. *The Lancet* 2017;**389**:815–22. doi:10.1016/S0140-6736(16)32401-1

2 Kasivisvanathan V, Rannikko AS, Borghi M, *et al.* MRI-Targeted or Standard Biopsy for Prostate-Cancer Diagnosis. *N Engl J Med* 2018;**378**:1767–77. doi:10.1056/NEJMoa1801993

3 Bass EJ, Pantovic A, Connor M, *et al.* A systematic review and meta-analysis of the diagnostic accuracy of biparametric prostate MRI for prostate cancer in men at risk. *Prostate Cancer Prostatic Dis* 2020;:1–16. doi:10.1038/s41391-020-00298-w

4 Radtke JP, Kuru TH, Boxler S, *et al.* Comparative analysis of transperineal template saturation prostate biopsy versus magnetic resonance imaging targeted biopsy with magnetic resonance imaging-ultrasound fusion guidance. *J Urol* 2015;**193**:87–94. doi:10.1016/j.juro.2014.07.098

5 Satapathy S, Singh H, Kumar R, *et al.* Diagnostic Accuracy of 68Ga-PSMA PET/CT for Initial Detection in Patients With Suspected Prostate Cancer: A Systematic Review and Meta-Analysis. *Am J Roentgenol* 2021;**216**:599–607. doi:10.2214/AJR.20.23912

6 Wright GL, Haley C, Beckett ML, *et al.* Expression of prostate-specific membrane antigen in normal, benign, and malignant prostate tissues. *Urol Oncol Semin Orig Investig* 1995;**1**:18–28. doi:10.1016/1078-1439(95)00002-Y

7 Bouchelouche K, Choyke PL. Advances in PSMA Positron Emission Tomography (PET) of Prostate Cancer. *Curr Opin Oncol* 2018;**30**:189–96. doi:10.1097/CCO.0000000000000439

8 Hoffmann MA, Wieler HJ, Baues C, *et al.* The Impact of 68Ga-PSMA PET/CT and PET/MRI on the Management of Prostate Cancer. *Urology* 2019;**130**:1–12. doi:10.1016/j.urology.2019.04.004

9 Kesch C, Kratochwil C, Mier W, *et al.* 68Ga or 18F for Prostate Cancer Imaging? *J Nucl Med* 2017;**58**:687–8. doi:10.2967/jnumed.117.190157

10 Albisinni S, Aoun F, Marcelis Q, *et al.* Innovations in imaging modalities for recurrent and metastatic prostate cancer: a systematic review. *Minerva Urol E Nefrol Ital J Urol Nephrol* 2018;**70**:347–60. doi:10.23736/S0393-2249.18.03059-X

11 De Visschere PJL, Standaert C, Fütterer JJ, *et al.* A Systematic Review on the Role of Imaging in Early Recurrent Prostate Cancer. *Eur Urol Oncol* 2019;**2**:47–76. doi:10.1016/j.euo.2018.09.010

12 Evangelista L, Zattoni F, Cassarino G, *et al.* PET/MRI in prostate cancer: a systematic review and meta-analysis. *Eur J Nucl Med Mol Imaging* 2021;**48**:859–73. doi:10.1007/s00259-020-05025-0

For peer review only - http://bmjopen.bmj.com/site/about/guidelines.xhtml

13 Moher D, Shamseer L, Clarke M, *et al.* Preferred reporting items for systematic review and meta-analysis protocols (PRISMA-P) 2015 statement. *Syst Rev* 2015;**4**:1. doi:10.1186/2046-4053-4-1

14 Ouzzani M, Hammady H, Fedorowicz Z, *et al.* Rayyan-a web and mobile app for systematic reviews. *Syst Rev* 2016;**5**:210. doi:10.1186/s13643-016-0384-4

15 Genetic landscape of prostate cancer conspicuity on multiparametric MRI: a protocol for a systematic review and bioinformatic analysis | BMJ Open. https://bmjopen.bmj.com/content/10/1/e034611.abstract (accessed 1 Mar 2021).

16 Whiting PF, Rutjes AWS, Westwood ME, *et al.* QUADAS-2: A Revised Tool for the Quality Assessment of Diagnostic Accuracy Studies. *Ann Intern Med* 2011;**155**:529–36. doi:10.7326/0003-4819-155-8-201110180-00009

17 Jones R, Cornford P. EAU and NICE guidelines for the diagnosis and management of prostate cancer. How wide is the channel? *J Clin Urol* 2018;**11**:149–53. doi:10.1177/2051415817720353

18 Dasgupta P, Davis J, Hughes S. NICE guidelines on prostate cancer 2019. *BJU Int* 2019;**124**:1–1. doi:https://doi.org/10.1111/bju.14815

19 Wise J. NICE recommends MRI for suspected prostate cancer to reduce biopsies. *BMJ* 2018;**363**:k5290. doi:10.1136/bmj.k5290

20 Emmett L, Leeuwen PJ van, Nandurkar R, *et al.* Treatment Outcomes from 68Ga-PSMA PET/CT–Informed Salvage Radiation Treatment in Men with Rising PSA After Radical Prostatectomy: Prognostic Value of a Negative PSMA PET. *J Nucl Med* 2017;**58**:1972–6. doi:10.2967/jnumed.117.196683

21 Grubmüller B, Baltzer P, Hartenbach S, *et al.* PSMA Ligand PET/MRI for Primary Prostate Cancer: Staging Performance and Clinical Impact. *Clin Cancer Res Off J Am Assoc Cancer Res* 2018;**24**:6300–7. doi:10.1158/1078-0432.CCR-18-0768

22 Kuten J, Fahoum I, Savin Z, *et al.* Head-to-Head Comparison of 68Ga-PSMA-11 with 18F-PSMA-1007 PET/CT in Staging Prostate Cancer Using Histopathology and Immunohistochemical Analysis as a Reference Standard. *J Nucl Med Off Publ Soc Nucl Med* 2020;**61**:527–32. doi:10.2967/jnumed.119.234187

For peer review only

Reporting checklist for protocol of a systematic review and meta analysis.

Based on the PRISMA-P guidelines.

Instructions to authors

Complete this checklist by entering the page numbers from your manuscript where readers will find each of the items listed below.

Your article may not currently address all the items on the checklist. Please modify your text to include the missing information. If you are certain that an item does not apply, please write "n/a" and provide a short explanation.

Upload your completed checklist as an extra file when you submit to a journal.

In your methods section, say that you used the PRISMA-Preporting guidelines, and cite them as:

Moher D, Shamseer L, Clarke M, Ghersi D, Liberati A, Petticrew M, Shekelle P, Stewart LA. Preferred Reporting Items for Systematic Review and Meta-Analysis Protocols (PRISMA-P) 2015 statement. Syst Rev. 2015;4(1):1.

		Reporting Item	Page Number
**Title**			
Identification	#1a	Identify the report as a protocol of a systematic review	1
Update	#1b	If the protocol is for an update of a previous systematic review, identify as such	N/A This protocol is not an update
**Registration**			
	#2	If registered, provide the name of the registry (such as PROSPERO) and registration number	3
**Authors**			
Contact	#3a	Provide name, institutional affiliation, e-mail address of all protocol authors; provide physical mailing address of corresponding author	1, 11
Contribution	#3b	Describe contributions of protocol authors and identify the guarantor of the review	11

For peer review only

**Amendments**			
	#4	If the protocol represents an amendment of a previously completed or published protocol, identify as such and list changes; otherwise, state plan for documenting important protocol amendments	N/A. It is not an amendment
**Support**			
Sources	#5a	Indicate sources of financial or other support for the review	11
Sponsor	#5b	Provide name for the review funder and / or sponsor	N/A Review is not funded
Role of sponsor or funder	#5c	Describe roles of funder(s), sponsor(s), and / or institution(s), if any, in developing the protocol	N/A Review is not funded
**Introduction**			
Rationale	#6	Describe the rationale for the review in the context of what is already known	4
Objectives	#7	Provide an explicit statement of the question(s) the review will address with reference to participants, interventions, comparators, and outcomes (PICO)	4
**Methods**			
Eligibility criteria	#8	Specify the study characteristics (such as PICO, study design, setting, time frame) and report characteristics (such as years considered, language, publication status) to be used as criteria for eligibility for the review	5
Information sources	#9	Describe all intended information sources (such as electronic databases, contact with study authors, trial registers or other grey literature sources) with planned dates of coverage	5
Search strategy	#10	Present draft of search strategy to be used for at least one electronic database, including planned limits, such that it could be repeated	10
Study records - data management	#11a	Describe the mechanism(s) that will be used to manage records and data throughout the review	5
Study records -	#11b	State the process that will be used for selecting studies (such	5

For peer review only

selection process		as two independent reviewers) through each phase of the review (that is, screening, eligibility and inclusion in meta-analysis)	
Study records - data collection process	#11c	Describe planned method of extracting data from reports (such as piloting forms, done independently, in duplicate), any processes for obtaining and confirming data from investigators	5
Data items	#12	List and define all variables for which data will be sought (such as PICO items, funding sources), any pre-planned data assumptions and simplifications	6
Outcomes and prioritization	#13	List and define all outcomes for which data will be sought, including prioritization of main and additional outcomes, with rationale	7
Risk of bias in individual studies	#14	Describe anticipated methods for assessing risk of bias of individual studies, including whether this will be done at the outcome or study level, or both; state how this information will be used in data synthesis	8
Data synthesis	#15a	Describe criteria under which study data will be quantitatively synthesised	7
Data synthesis	#15b	If data are appropriate for quantitative synthesis, describe planned summary measures, methods of handling data and methods of combining data from studies, including any planned exploration of consistency (such as I2, Kendall's τ)	7
Data synthesis	#15c	Describe any proposed additional analyses (such as sensitivity or subgroup analyses, meta-regression)	7
Data synthesis	#15d	If quantitative synthesis is not appropriate, describe the type of summary planned	N/A Quantitative synthesis is appropriate
Meta-bias(es)	#16	Specify any planned assessment of meta-bias(es) (such as publication bias across studies, selective reporting within studies)	7
Confidence in cumulative evidence	#17	Describe how the strength of the body of evidence will be assessed (such as GRADE)	7

For peer review only

Notes:

- 1b: N/A This protocol is not an update
- 4: N/A. It is not an amendment
- 5b: N/A Review is not funded
- 5c: N/A Review is not funded
- 15d: N/A Quantitative synthesis is approprirate The PRISMA-P elaboration and explanation paper is distributed under the terms of the Creative Commons Attribution License CC-BY. This checklist was completed on 07. April 2021 using https://www.goodreports.org/, a tool made by the EQUATOR Network in collaboration with Penelope.ai

For peer review only
